# Ultrasound evaluation of epicardial fat and *Eco-Obesity* body composition assessment

**DOI:** 10.3389/fendo.2025.1746253

**Published:** 2026-01-15

**Authors:** Silvana di Gregorio, Eduardo Blanco, Marta Calbo, Olga Rossell, Laia Dachs, Júlia Bonet, Andrea Jover, Lidia Huanuco, Marcos Yañez, Irina Faja, Francisco de Cabo, Guillem Cuatrecasas

**Affiliations:** 1 Grup CPEN Endocrinologia i Nutrició, Barcelona, Spain; 2 Centre Mèdic Vic, Barcelona, Spain; 3 Servicio Cardiologia Hospital Teknon, Grupo Quiron Salud, Barcelona, Spain; 4 Unidad Multidisciplinaria de Obesidad Hospital Teknon, Grupo Quiron Salud, Barcelona, Spain; 5 Institut Guirado Radiologia, Barcelona, Spain; 6 Faculty Health Sciences, Open University Catalonia, Barcelona, Spain

**Keywords:** body composition analysis, cardiovascular risk, DEXA-scan, echocardiography, Eco-Obesity, epicardial adipose tissue, obesity, ultrasound

## Abstract

**Objectives:**

To validate echocardiographic epicardial adipose tissue (EAT) thickness as a non-invasive marker of cardiometabolic risk in obesity and compare Eco-Obesity ultrasound-derived measurements of epicardial, preperitoneal, omental, and peri-renal fat depots with standard body composition analysis by dual-energy X-ray absorptiometry (DEXA).

**Methods:**

In a cross-sectional study of 402 adults with obesity attending two Barcelona centers, Eco-Obesity ultrasound was performed in 114 subjects, alongside DEXA scans. Thickness of epicardial and abdominal fat depots, metabolic markers, and anthropometric indices were measured using standardized protocols. Relationships among ultrasound fat measurements, DEXA indices, anthropometric variables, and biochemical markers were analyzed by Pearson correlation coefficients; subgroup comparisons used ANOVA.

**Results:**

Moderate (>7mm) and severe (>10mm) EAT thickness were found in 35.6% and 53.1% of patients. EAT measured in long-axis, short-axis, and posterior cardiac recess (PPR) projections correlated significantly with BMI, waist circumference, and waist-to-height ratio (all p<0.02), and was associated with omental and peri-renal fat compartments. The EAT long-axis projection showed the strongest and most consistent correlations with anthropometric and DEXA visceral fat indices. Omental and peri-renal fat layers correlated significantly with anthropometric and DEXA visceral fat, while pre-peritoneal fat did not. EAT PPR correlated positively with glycemia, HOMA-IR, triglycerides, and negatively with HDL cholesterol; EAT long- and short-axis also correlated with glycemia and HOMA-IR. Omental fat exhibited the strongest associations with glycemia, HOMA index, and HbA1c, and peri-renal fat showed significant positive association with fasting glycemia and inverse association with HDL.

**Conclusions:**

EAT is a metabolically active visceral depot associated with adiposity and metabolic dysfunction in obesity. Routine ultrasonographic assessment of EAT should be integrated into clinical protocols for body composition analysis in the obese population (Eco-Obesity approach).

## 1 Introduction

Obesity is a chronic, complex metabolic disease (1) characterized by increased total and *ectopic* fat depots, some of them (*omental* and *peri-renal* fat) with a strong and well-established association with heightened cardiovascular morbidity and mortality (2, 3). Epicardial adipose tissue (EAT), an anatomically contiguous fat depot, is primarily distributed in atrioventricular and interventricular grooves and along the coronary arteries, enveloping the surface of the heart. EAT is precisely defined as the adipose space between the external myocardial wall and the visceral layer of the pericardium. Its unique anatomic, metabolic, and paracrine features are implicated in the progression of coronary artery disease (CAD), atrial fibrillation, and heart failure. Notably, pathological EAT expansion represents an independent predictor of incident metabolic syndrome in men (4), and increased EAT thickness off >5 mm provides clinically relevant discrimination for significant asymptomatic and established CAD (5).

Mechanistically, the pathogenicity of epicardial fat extends beyond passive lipid storage, as this depot demonstrates pronounced metabolic activity and secretes a range of adipocytokines, hormones, and vasoactive peptides known to modulate vascular and myocardial function (6). Increases in EAT and lipomatous hypertrophy (massive deposits of fat within the atrial septum) are associated with arrhythmogenic risk, enhanced coronary calcification, and adverse post-infarction outcomes (7, 8). Together with other visceral fat compartments, epicardial adipose tissue’s endocrine functions contribute acutely to the development of a proatherogenic, inflammatory microenvironment within the adjacent myocardium and coronary vasculature (7, 8).

Current cardiovascular imaging modalities such as computed tomography (CT) and magnetic resonance imaging have been used for volumetric EAT assessment (5, 7, 8), but high cost and repeated radiation limits its accessibility. In contrast, ultrasonography allows reliable measurement of EAT thickness, in a non-radiative, widely and cost-effective way (5, 9). Echo-identified EAT appears as the echo-free space between the outer myocardium and visceral pericardium, with thickness measurements typically ranging from 5 mm up to 23 mm in severely affected individuals (9). Importantly, echocardiographic EAT thickness correlates more closely with markers of visceral adiposity and strongly associates with metabolic syndrome (4), insulin resistance, subclinical atherosclerosis, and risk for future cardiovascular events (9). Its measurement also dynamically responds to weight loss interventions, paralleling reductions in metabolic risk (3, 10).

Studies have shown that, among all visceral depots, *omental* and *peri-renal* fat measurements, are associated with metabolic syndrome and fatty liver risk, and specific thickness cut-offs may enhance risk classification (5, 11, 12). Echographic EAT assessment can be done as a routine procedure within the global ultrasound body composition assessment in patients with obesity (*Eco-Obesity*) (11), enabling non-invasive, simultaneous quantification of epicardial and multiple abdominal fat compartments, including superficial and profound *subcutaneous*, *pre-peritoneal*, *omental*, and *peri-renal* depots, which are not reliably distinguished by Dual-energy X-ray absorptiometry (DEXA), CT or even MNR.

Recent clinical guidelines now explicitly categorize obesity via both anthropometric and clinical criteria. We therefore need new tools for advanced body composition assessment (such as sonography) to phenotype obesity and redefine cardiovascular risk stratification (1, 3). These guidelines advocate moving beyond traditional body mass index (BMI) dependence, highlighting the diagnostic importance of regional and ectopic adipose depots, and supporting the integration of non-invasive markers into risk prediction, patient staging, and individualized management paradigms (1, 3).

Despite increasing recognition of the role of EAT in cardiovascular and metabolic risk, significant knowledge gaps persist regarding its quantitative and functional relationships with overall and other ectopic adipose depots. This study aims to characterize EAT fat compartments measured in three regions (long-axis, short-axis and posterior cardiac (pericardial) recess [PPR]), in a large cohort of patients with obesity, as part of a standardized *Eco-Obesity* procedure. Secondary objectives are correlations between *Eco-Obesity* ultrasound-measured ectopic fat compartments (including EAT and other cardiovascular risk-associated fat compartments [*pre-peritoneal*, *omental* and *peri-renal* layers]) and body fat composition obtained using gold-standard DEXA, anthropometric and biochemical metabolic parameters.

## 2 Methods

### 2.1 Study design and subjects

Cross-sectional study evaluating n=402 consecutive patients with obesity (BMI ≥30 kg/m²) attending the Unidad de Obesidad Cardio-Metabolica, Centre Mèdic Vic (Barcelona, Spain) between January and September 2025. All participants underwent a clinical evaluation, which included detailed anthropometric measurements such as weight, height, BMI, waist circumference, and waist-to-height ratio measurements. Clinically relevant thresholds were applied for cardiovascular risk assessment: abnormal waist-to-height ratio >0.5 indicating increased central adiposity and cardiovascular risk, with values ≥0.58 associated with significantly elevated mortality risk from cardiovascular disease, diabetes, and other obesity-related conditions (13). BMI classifications followed standard criteria (normal: 18.5-24.9 kg/m², overweight: 25-29.9 kg/m², obese: ≥30 kg/m²). Waist circumference thresholds for abdominal obesity were defined as >102 cm in men and >88 cm in women.

Comorbidities were assessed as follows: type 2 diabetes was diagnosed according to ADA guidelines (14). Abnormal blood pressure was defined as a 3-times measured value >130 mm Hg for systolic and/or >85 mm Hg for diastolic blood pressure and dyslipidemia was defined as serum triglycerides (TGs) >150 mg/dL in women and >200 mg/dL in men, or high-density lipoprotein (HDL) cholesterol <40 mg/dL in men and <50 mg/dL in women (15). Dyslipidemic patients with known carotid atheromatosis (CA) were also recorded. As part of routine screening, all patients received a standard electrocardiogram (ECG) to document baseline cardiac electrical activity and detect any previously undiagnosed cardiac conditions. Patients with ECG abnormalities and known CAD were not included in the analysis.

Biochemical parameters were analyzed from fasting blood samples. The panel included glycemia, glycated hemoglobin (HbA1c), serum insulin (enzyme immunoanalysis ALINITY C reagents^©^ (Abbot Diagnostics) with 3 μU/mL sensibility), and calculation of the homeostatic model assessment for insulin resistance (HOMA-IR). Lipid profiling incorporated HDL, low density lipoprotein (LDL) and triglycerides (TG). All measurements followed standardized protocols and were performed by experienced staff.

### 2.2 Echocardiographic assessment of EAT and other cardiovascular risk-associated fat compartments

**Figure 1:**
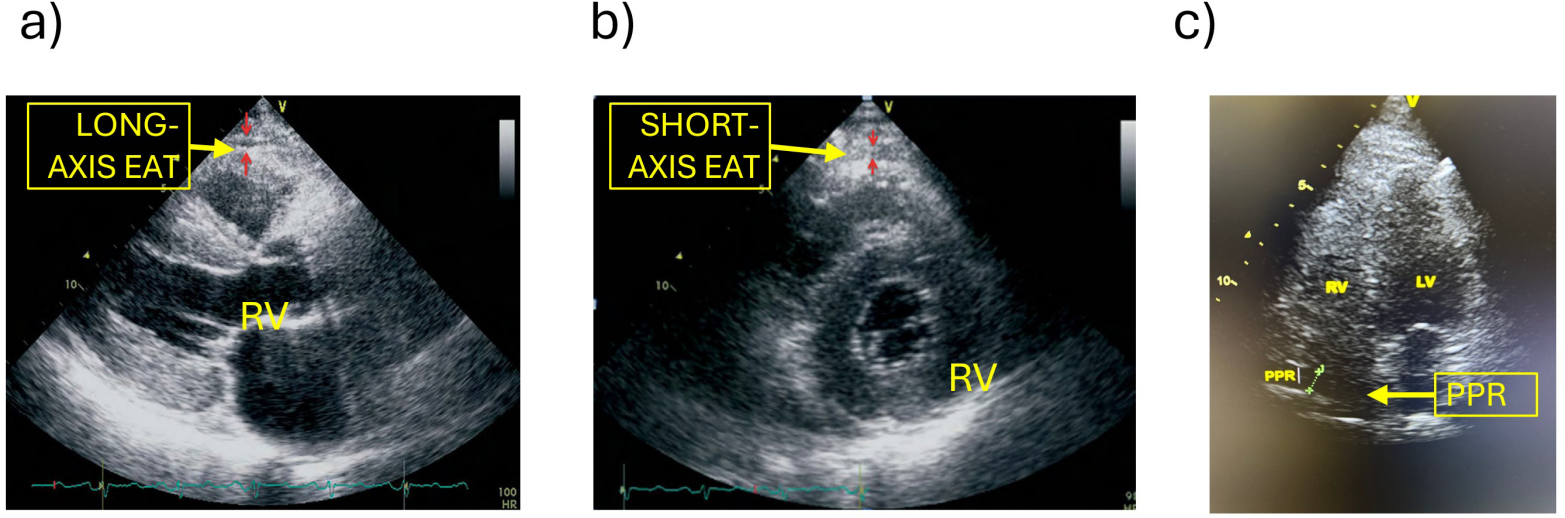
Ultrasound images corresponding to the three EAT measurements **(a)** long-axis, **(b)** short-axis, and **(c)** PPR. EAT, Epicardial adipose tissue; LV, left ventricle; PPR, posterior cardiac (pericardic) recess; RV, right ventricle. .

Transthoracic echocardiography was performed using a Vivid 8 ultrasound system (GE Healthcare^®^) with a 2.5–5 MHz phased-array transducer. All cardiac examinations (including cardiac ultrasound assessment of EAT) were conducted by an experienced cardiologist following a standardized protocol to minimize inter-observer variability. EAT is non-homogeneously distributed around the heart, being predominantly located at the cardiac base, apex, in the cardiac grooves, and around the coronary arteries, with greatest thickness around the right ventricle compared to the left (16). For this reason, and in accordance with current recommendations, EAT thickness was assessed in three regions: the parasternal long-axis, parasternal short-axis, and posterior cardiac (pericardial) recess (PPR), two of which were previously described by Iacobellis et al. (5), and a third innovative echocardiographic plane corresponding to the PPR. The long-axis and short-axis views in 2D enabled more accurate measurement of epicardial fat thickness over the right ventricle. Thickness was measured perpendicularly over the free wall of the right ventricle at end-systole across three consecutive cardiac cycles. The aortic annulus served as an anatomical reference point for consistent localization of the ventricular wall. Reproducibility of the measurements obtained in end-diastole on the free wall of the right ventricle (parasternal long-axis view) showed excellent intraobserver (ICC = 0.98; CV = 3.0–3.5%) and interobserver agreement (ICC = 0.96; CV = 5.0–5.5%). Measurement of epicardial fat within the PPR by transthoracic echocardiography was implemented to enable quantification of the adipose tissue located between the epicardium and pericardium in this specific anatomical space, representing a novel approach for clinical application. The PPR view is distinguished from conventional projections, providing a complementary assessment of EAT distribution. This projection is typically obtained via a subxiphoid echocardiographic approach. Briefly, in the four-chamber echocardiographic view, the PPR appears as an anechoic zone posterior to the right atrium, between the free wall of the right atrium and the pericardium (**Figure 1**). Measurement was performed at the point of maximal EAT thickness, in millimeters at end-systole, averaging three cardiac cycles to reduce variability. All measurements were recorded, and additional findings such as pericardial effusion or ventricular anomalies were noted. The measured values were compared to reference cut-offs for cardiovascular risk stratification (17).

Epicardial fat thickness severity was categorized according to established criteria: normal: <5 mm, mild: 5–7 mm, moderate: 7–10 mm, and severe: >10 mm (17). Measurements were performed during suspended respiration to minimize respiratory artifacts.

### 2.3 Body composition assessment

Body composition analysis was performed using dual-energy X-ray absorptiometry with a Lunar Prodigy DEXA scanner (GE Healthcare, Software version 18). DEXA measurements included total body fat mass, regional fat distribution, and visceral fat estimation using the CoreVat algorithm, which serves as the gold-standard for visceral adiposity quantification (which corresponds to the sum of *pre-peritoneal, omental*, and *peri-renal*). Body fat percentages above 25% in men and 32% in women may indicate obesity-related cardiometabolic risk (1).

Standardized anthropometric assessments included body weight (kg), height (cm), BMI (kg/m²), waist circumference (cm), and waist-to-height ratio. All measurements were performed by trained personnel following international standardized protocols.

A subgroup of patients (n=114) were submitted (Obesity Unit of the CPEN Group for Endocrinology and Nutrition (Barcelona, Spain) to a standardized *Eco-Obesity* ultrasound evaluation to routinely assess abdominal fat layers with metabolic and cardiovascular relevance: superficial *subcutaneous*, deep *subcutaneous*, *pre-peritoneal*, *omental* and *peri-renal* fat thickness, following the protocol defined by Cuatrecasas et al. (10). Briefly, when comparing DEXA with *Eco-Obesity* ultrasound, the measurement should be performed at the L4 level, rather than at any level referenced to the umbilicus (18), which corresponds ultrasonographically to the bifurcation of the iliac arteries. These measurements were correlated with epicardial fat thickness.

### 2.4 Statistical analyses

Statistical analyses were performed using SPSS version 23. Continuous variables were expressed as mean ± standard deviation, and categorical variables as frequencies and percentages. Analysis of variance (ANOVA) was applied to compare group means and to identify differences across subgroups. When applicable, *post hoc* comparisons were conducted to determine pairwise differences. Pearson correlation coefficients (R) were calculated to assess relationships between EAT, DEXA-derived visceral fat measurements, and biochemical parameters. A p-value <0.05 was considered statistically significant. DEXA measurements were performed according to manufacturer specifications with regular calibration protocols.

### 2.5 Ethics statement

The study was conducted in accordance with the principles expressed in the Declaration of Helsinki, and the protocol followed the Good Clinical Practice guidelines and was approved by the Ethical Committee of Hospital Teknon-Grupo Quiron Salud, Barcelona. Given the retrospective nature of this study using data from routine clinical practice, general informed consent for research use of anonymized clinical data was obtained from all participants.

## 3 Results

### 3.1 Demographic characteristics

The study included a total of 402 patients in the overall cohort, with a dedicated subgroup of 114 participants undergoing detailed *Eco-Obesity* ultrasound body composition evaluation. In the overall cohort, 68.8% were female (n=277) and 31.1% male (n=125). The mean age was 55.3 years (SD = 11.8) for women and 53.3 years (SD = 13.1) for men, and 57.2% of female participants were postmenopausal. In the subgroup, 58.8% were female (n=67) and 41.2% male (n=47). The mean age was 57.6 years (SD = 11.5) for women and 54.7 years (SD = 10.5) for men, and 77.6% of female participants were postmenopausal (**Table 1**).

**Table 1:** Demographic and anthropometric characteristics of participants.

	Overall cohort (n=402)		*Eco-Obesity* ultrasound evaluation subgroup (n=114)	
	Females	Males	Females	Males
Participants	277 (68.8)	125 (31.1)	67 (58.8)	47 (41.2)
Age, years	55.3 (11.8)	53.3 (13.1)	57.6 (11.5)	54.7 (10.5)
Post menopause, n (%)	158 (57.2)	–	50 (43.9)	–
BMI, kg/m^2^	34.4 (5.6)	34.6 (6.1)	32.8 (5.3)	33.6 (5.8)
Percentage of body fat	47.9 (4.8)	39.4 (5.9)	48.8 (5.1)	39.2 (4.9)
Waist	101.5 (10.9)	103.7 (13.1)	105.1 (12.1)	115.9 (9.9)
Waist-to-height	0.6 (0.1)	0.7 (0.1)	0.7 (0.1)	0.7 (0.1)
Diabetes, n (%)	19 (8.5)	22 (17.3)	1 (1.5)	4 (8.5)
Hypertension, n (%)	58 (25.4)	54 (42.9)	20 (29.9)	24 (51.1)
Dyslipidemia, n (%)	47 (20.8)	41 (32.6)	20 (29.9)	19 (40.4)
CA, n (%)	16 (5.7)	23 (18.4)	3 (4.5)	12 (25.5)

Results are expressed as mean (standard deviation) unless specified.

BMI, Body mass index; CA, Carotid atheromatosis.

Anthropometric characteristics in the overall cohort are presented in **Table 1**. Overall, females showed a mean BMI of 34.4 kg/m², mean body fat percentage of 47.9%, mean waist circumference of 101.5 cm, and mean waist-to-height ratio of 0.64, and males showed values of 34.7 kg/m², 39.4%, 103.7 cm, and 0.65, respectively. Characteristics were similar in the *Eco-Obesity* subgroup.

Prevalence of all comorbidities analyzed (diabetes, hypertension, dyslipidemia, and carotid atheromatosis) was higher in men than in women both in the overall cohort and in the *Eco-Obesity* subgroup (**Table 1**).

### 3.2 Characterization of EAT and other cardiovascular risk-associated fat compartments

Among the 143 patients assessed, only one individual (0.9%) exhibited normal EAT thickness (<5 mm), while 15 participants (10,4%) presented with low-thickness (5–7 mm), 51 (35,6%) with moderate thickness (7–10 mm), and the majority, 76 (53,1%), with severe EAT thickness (>10 mm) (**Table 2**).

**Table 2:** Characterization of EAT layers measured by *Eco-Obesity*.

	Females	Males
Severity of EAT fat thickness		
Normal (< 5 mm), n (%)	1 (0.5)	0 (0.0)
Low (5–7 mm), n (%)	15 (8.2)	1 (2.1)
Moderate (7–10 mm), n (%)	51 (27.7)	14 (29.8)
Severe (>10 mm), n (%)	76 (41.3)	32 (68.1)
EAT Long-axis	10.0 (2.4)	11.5 (2.4)
EAT Short-axis	9.6 (2.4)	10.7 (2.9)
EAT PPR	9.6 (2.6)	10.9 (2.8)

Results are expressed as mean (standard deviation) unless specified.

EAT, Epicardial adipose tissue; PPR, posterior cardiac (pericardiac) recess.

Sex-stratified analysis revealed consistently higher EAT measurements in males across all anatomical projections. In the parasternal long-axis view, mean EAT thickness was 11.5 mm (SD 2.4) in males versus 10.0 mm (SD 2.4) in females. Similar trends were observed in the short-axis (10.7 mm [SD 2.9] in males vs. 9.6 mm [SD 2.4] in females) and posterior cardiac recess (PPR) projections (10.9 mm [SD 2.8] in males vs. 9.6 mm [SD 2.6] in females) (**Table 2**).

### 3.3 Correlation between EAT and other cardiovascular risk-associated fat compartments, DEXA and anthropometric measurements

The EAT long-axis projection exhibited the most consistent associations across weight, BMI, waist circumference, waist-to-height ratio, and total body fat (R values 0.18–0.37, all p<0.01), while the PPR projection showed the highest individual correlation only with weight (R = 0.39, p<0.001). Short-axis EAT was significantly related to most variables but did not correlate with waist circumference in females or with total body fat. Correlations between EAT thickness in these projections and DEXA-derived visceral adipose tissue (VAT) were all statistically significant but modest in strength (long-axis R = 0.37, short-axis R = 0.32, PPR R = 0.36; all p<0.001; **Tables 3**, **4**).

**Table 3:** Correlation between EAT, other cardiovascular risk-associated fat compartments, DEXA and anthropometric measurements.

Eco-Obesity	Weight	BMI	Waist (F)	Waist (M)	Waist/Height index	Total body fat (kg)
EAT						
EAT Long-axis	0.321 (<0.001)	0.305 (<0.001)	0.176 (0.02)	0.370 (<0.001)	0.284 (<0.001)	0.280 (<0.001)
EAT Short-axis	0.182 (<0.001)	0.199 (<0.001)	NS	0.415 (<0.001)	0.258 (<0.001)	NS
EAT PPR	0.389 (<0.001)	0.294 (<0.001)	0.212 (<0.001)	0.310 (<0.001)	0.240 (<0.001)	0.292 (<0.001)
Other cardiovascular risk-associated fat compartments						
*Omental* layer	0.488 (<0.001)	0.417 (<0.001)	0.466 (<0.001)	0.404 (<0.001)	0.443 (<0.001)	0.302 (0.05)
*Peri-renal* layer	0.387 (<0.001)	0.238 (0.05)	NS	NS	0.423 (<0.001)	0.176 (0.05)
*Pre-peritoneal* layer	NS	NS	NS	NS	NS	NS
DEXA						
VAT	0.553 (<0.001)	0.384 (<0.001)	0.656 (<0.001)	0.591 (<0.001)	0.561 (<0.001)	0.358 (<0.001)

Results are presented as R (p-value). Statistically significant correlations are shown in bold.

BMI, Body mass index; DEXA, Dual-energy X-ray absorptiometry; EAT, Epicardial adipose tissue; F, Females; PPR, posterior cardiac (pericardic) recess; M, males; NS, Non-significant; R, Pearson coefficients; VAT, Visceral abdominal fat.

**Table 4:** Correlation between EAT and other cardiovascular risk-associated fat compartments and body fat composition with DEXA.

	Visceral Fat Mass measured by DEXA -VAT-	*Omental* Layer measured by *Eco-Obesity*
EAT		
EAT Long-axis	0.370 (<0.001)	0.313 (0.002)
EAT Short-axis	0.323 (<0.001)	0.336 (<0.001)
EAT PPR	0.364 (<0.001)	0.459 (<0.001)
Other cardiovascular risk-associated fat compartments		
*Omental* layer	0.644 (<0.001)	–
*Peri-renal* layer	0.659 (<0.001)	0.582 (<0.001)

Results are presented as R (p-value).

DEXA, Dual-energy X-ray absorptiometry; EAT, Epicardial adipose tissue; PPR, posterior cardiac (pericardic) recess; R, Pearson coefficients; VAT, Visceral abdominal fat.

Assessment of other cardiovascular risk-associated fat compartments showed that *omental* fat layer was most strongly correlated with body weight, BMI, waist circumference, and waist-to-height ratio for both sexes (all p<0.001), with the highest correlations observed for waist circumference (R = 0.466 in females; R = 0.404 in males) and waist-to-height ratio (R = 0.443, p<0.001). *Peri-renal* fat also correlated significantly with weight (R = 0.387, p<0.001), BMI (R = 0.238, p<0.05), waist-to-height ratio (R = 0.423, p<0.001), and total body fat (R = 0.176, p<0.05). In contrast, the *pre-peritoneal* layer showed no significant associations with anthropometric or body composition parameters.

DEXA-derived VAT also presented strong positive correlations with all anthropometric measurements and with *omental* fat layer (R = 0.64, p<0.001) and *peri-renal* fat (R = 0.66, p<0.001) (**Tables 3**, **4**).

### 3.4 Correlation between EAT and other cardiovascular risk-associated fat compartments

**Figure 2:**
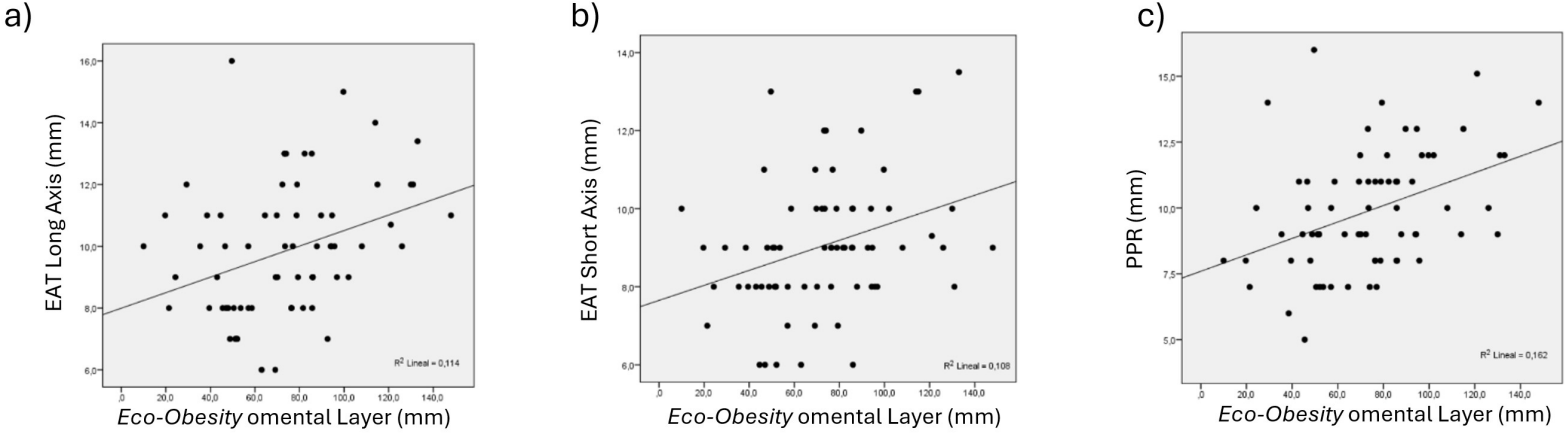
Correlation between EAT measurements **(a)** long-axis, **(b)** short-axis, and **(c)** PPR, and *omental* layer, all measured by *Eco-Obesity*. EAT, Epicardial adipose tissue; PPR, posterior cardiac (pericardic) recess.

**Figure 3:**
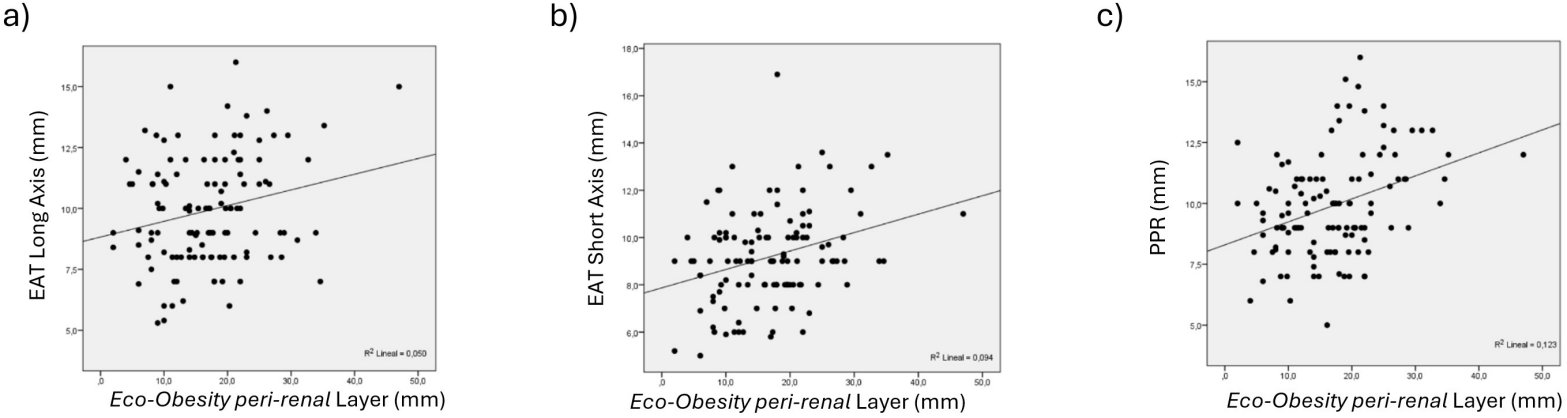
Correlation between EAT measurements **(a)** long-axis, **(b)** short-axis, and **(c)** PPR, and *peri-renal* layer, all measured by *Eco-Obesity*. EAT, Epicardial adipose tissue; PPR, posterior cardiac (pericardic) recess.

**Figure 4:**
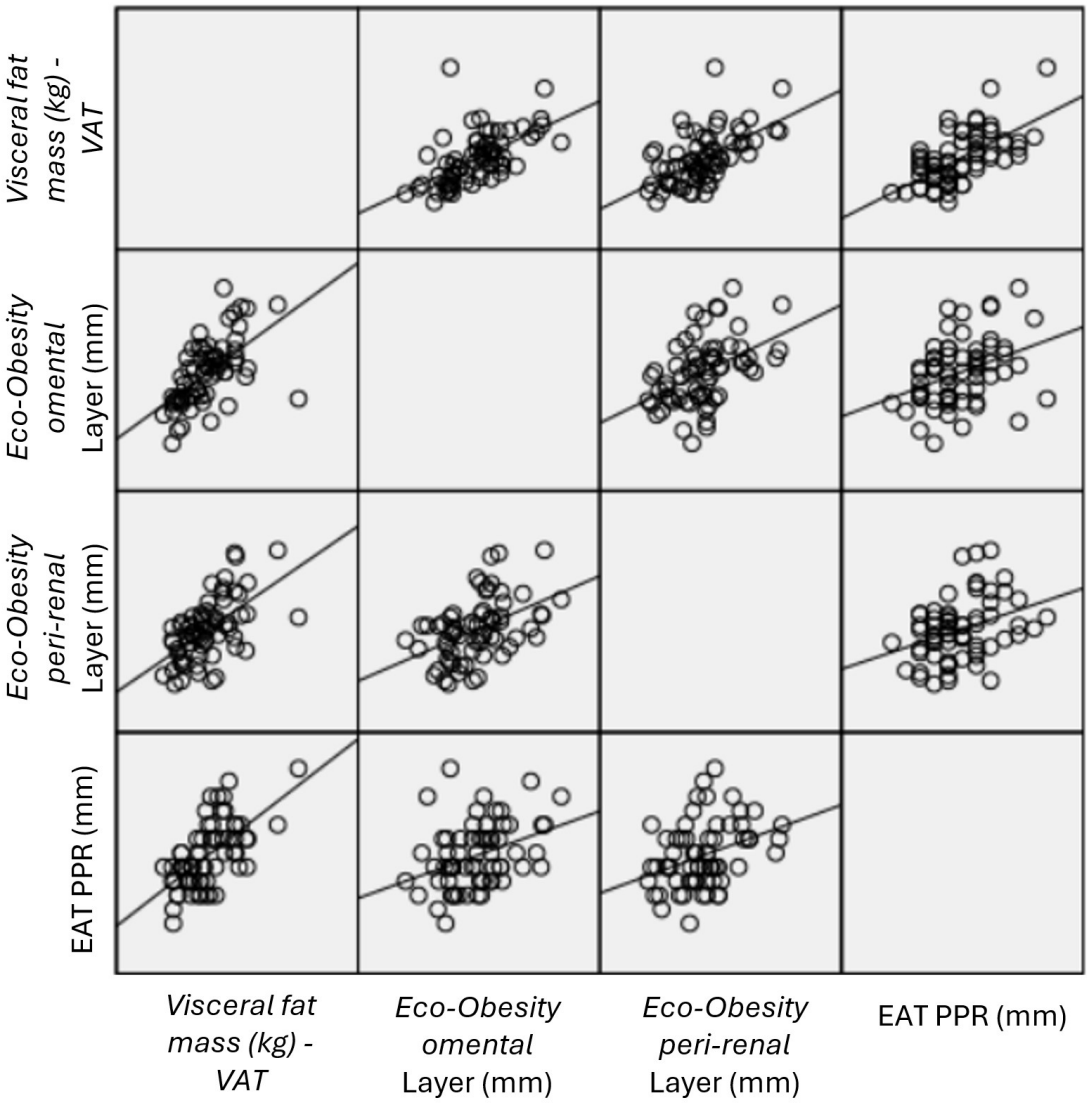
Correlations between EAT PPR, *omental* layer and *peri-renal* layer, all measured by *Eco-Obesity*, and VAT measured by DEXA. DEXA, Dual-energy X-ray absorptiometry; EAT, Epicardial adipose tissue; PPR, posterior cardiac (pericardic) recess; VAT, Visceral abdominal fat. .

Correlation analyses demonstrated significant associations between EAT thickness and other cardiovascular risk-associated abdominal fat compartments, all evaluated by *Eco-Obesity* ultrasound. EAT thickness measured in long-axis, short-axis, and PPR views presented moderate correlations with *omental* (**Figure 2**) and *peri-renal* (**Figure 3**) fat thickness, with R values ranging from 0.31 to 0.58 (all p<0.002). Specifically, strongest association was observed for PPR views and *omental* layer (R = 0.46, p<0.001) and *peri-renal* layer (R = 0.58, p<0.001) (**Table 4**, **Figure 4**).

### 3.5 Association Between EAT and other cardiovascular risk-associated fat compartments and biochemical metabolic parameters

Regarding EAT, both long and short-axis showed weak but significant correlations with glycemia and HOMA index, though no associations were found with HbA1c or lipid variables. The PPR view of EAT was positively correlated with glycemia (R = 0.128, p=0.021) and HOMA index (R = 0.134, p=0.033), and also showed a significant negative association with HDL cholesterol (R=-0.116, p=0.042) and a positive association with triglycerides (R = 0.127, p=0.024) (**Table 5**), but no association with LDL.

**Table 5:** Association between EAT, other cardiovascular risk-associated fat compartments, DEXA and biochemical metabolic parameters.

Eco-Obesity	Glycemia (mg/dl)	HOMA-IR index	HbA1c (%)	HDL (mg/dl)	TG (mg/dl)
EAT					
EAT long-axis	**0.117 (0.034)**	**0.184 (0.003)**	0.056 (0.342)	-0.064 (0.259)	0.063 (0.264)
EAT short-axis	**0.139 (0.012)**	**0.131 (0.036)**	0.104 (0.075)	-0.015 (0.797)	0.054 (0.340)
EAT PPR	**0.128 (0.021)**	**0.134 (0.033)**	0.106 (0.073)	**-0.116 (0.042)**	**0.127 (0.024)**
Other cardiovascular risk-associated fat compartments					
*Omental* layer	**0.380 (<0.001)**	**0.279 (0.012)**	**0.232 (0.021)**	-0.187 (0.060)	0.133 (0.183)
*Peri-renal* layer	**0.218 (0.010)**	0.021 (0.832)	0.073 (0.402)	**-0.221 (0.010)**	0.021 (0.678)
*Pre-peritoneal* layer	NS	NS	NS	NS	NS
DEXA					
VAT	**0.302 (<0.001)**	**0.316 (<0.001)**	**0.132 (0.018)**	**0.210 (<0.001)**	**0.220 (<0.001)**

Statistically significant correlations are shown in bold.

DEXA, Dual-energy X-ray absorptiometry; EAT, Epicardial adipose tissue; PPR, posterior cardiac (pericardic) recess; HbA1c, glycated hemoglobin; HDL, high-density lipoprotein; HOMA, homeostatic model assessment for insulin resistance; P, p-value; R, Pearson’s R; TG, triglycerides; VAT, Visceral abdominal fat.

The *omental* fat layer exhibited the strongest metabolic associations among the abdominal ectopic fat depots, demonstrating significant positive correlations with glycemia (R = 0.380, p<0.001), HOMA index (R = 0.279, p=0.012), and HbA1c (R = 0.232, p=0.021). The only trend with HDL cholesterol was a weak and non-significant inverse relationship (R=-0.187, p=0.060), while correlations with triglycerides were not significant. *Peri-renal* fat demonstrated a modest but significant positive association with fasting glycemia (R = 0.218, p=0.010) and an inverse association with HDL cholesterol (R=-0.221, p=0.010). No significant associations were observed between *pre-peritoneal* fat and metabolic parameters (**Table 5**).

## 4 Discussion

Our main findings reinforce the concept that EAT is a major metabolically active visceral fat depot, closely related to markers of adiposity and metabolic dysfunction in patients with obesity. CT and MRN data did not show the striking prevalence of moderate and severe EAT thickness observed in our study. This comparison with DEXA results provides the first direct, head-to-head validation of a global *Eco-Obesity* ultrasound (with EAT measurement as part of the protocol) measurements against a recognized gold-standard for body composition assessment. EAT and all other ectopic depots correlated significantly with VAT obtained from DEXA. Another important finding is the significant association between EAT thickness and the waist-to-height ratio, an emerging anthropometric index increasingly recognized as a simple marker of visceral adiposity and cardiovascular risk.

All three echocardiographic projections of EAT showed consistent correlations with parameters of glucose metabolism, lipid profile, and insulin resistance, with PPR showing the strongest association. Also, the correlations identified between *omental* and *peri-renal* fat are consistent with their recognized roles as the principal visceral adipose compartments linked to cardiovascular risk; these compartments should thus be prioritized in ultrasound-based phenotyping of obesity due to their established metabolic and prognostic significance for cardiovascular health.

The correlations seen in this study are consistent with previous reports linking intra-abdominal fat layers to cardiometabolic risk but further highlights the role of the waist-to-height ratio, a variable frequently used in primary care screening. The waist-to-height threshold of 0.5, widely adopted in preventive medicine, identifies individuals with greater cardiometabolic risk and higher EAT values, and highlights the potential of combining echocardiographic and anthropometric assessments to refine phenotyping of obesity beyond BMI, as recommended by recent clinical guidelines (1, 3). The positive association between ultrasound-measured *omental* fat and total body fat quantified by DEXA (R = 0.302, p=0.05) also supports the reliability of *Eco-Obesity* ultrasonography as a practical method for assessing visceral adiposity in clinical research. Additionally, it is essential to mention that *Eco-Obesity* ultrasonography is the only technique capable of stratifying the different fat layers, highlighting its clinical usefulness.

When analyzing ectopic fat compartments, the study confirms that *omental* and *peri-renal* fat, but not *pre-peritoneal* fat, correlate with both EAT thickness and the metabolic parameters defining insulin resistance and dyslipidemia. The absence of significant associations involving *pre-peritoneal* fat, neither with anthropometric variables, DEXA-derived VAT, nor EAT, demonstrates that *pre-peritoneal* adiposity is metabolically irrelevant despite its technical accessibility. This observation challenges the accepted assumption that *pre-peritoneal* fat correlates with visceral adiposity and could be an easy-to-do assessment of cardiovascular risk estimation and points out to the need to prioritize *omental* and *peri-renal* targets for accurate visceral fat quantification. These findings confirm the usefulness of ultrasound-guided visceral fat analysis and emphasize the greater metabolic relevance of *omental* and *peri-renal* depots compared to the *pre-peritoneal* fat compartment. To our opinion, when addressing cardiovascular and cardiometabolic risk phenotyping in patients with obesity, we should focus on these 3 ultrasound measurements: *epicardial*, *omental* and *peri-renal*, rather than more superficial depots (*subcutaneous* and *pre-peritoneal*). These findings confirm and expand earlier observations that ultrasonography, when standardized, can provide a practical and reproducible clinical tool for simultaneous quantification of cardiac and abdominal visceral fat (11, 19, 20).

Furthermore, the relationship with glycemia, HbA1c, HDL, triglycerides, and HOMA index, but not LDL suggests that EAT accumulation is closely linked to insulin resistance, as reflected by analogous behavior of key abdominal visceral fat depots. Our results demonstrate that the *omental* layer shows the strongest associations with glycemic parameters and indices of insulin resistance, while *peri-renal* fat shows significant but weaker metabolic correlations. These findings align with previous data (11) but reveal stronger associations, which demonstrates a metabolic interdependence between cardiac and abdominal ectopic fat compartments.

Recent data further support a tight interplay between epicardial fat, whole-body composition, and muscle function (21, 22). Epicardial adipose tissue behaves as a metabolically active visceral depot that correlates with anthropometric indices, DEXA-derived visceral fat and cardiometabolic traits, and is now considered a relevant imaging biomarker within ultrasound-based body composition phenotyping in obesity. In parallel, CT- and echocardiography-based studies consistently show that low muscle quantity and quality (e.g. reduced psoas area or handgrip strength as a proxy of global muscle function) are independently associated with higher epicardial fat burden, adverse cardiovascular events, and mortality across cardiac and critical-care populations (23). Moreover, the inverse, age-independent relationship between muscle strength and epicardial fat thickness in healthy adults suggests that simple functional tests such as handgrip strength may help to identify subjects with excessive epicardial fat accumulation and increased cardiovascular risk, bridging the gap between structural (fat depots) and functional (sarcopenia/dynapenia) aspects of body composition (21). We did not include DEXA-derived indices of muscle mass or direct measures of muscle strength, and therefore the correlation between epicardial adipose tissue and skeletal muscle could not be evaluated in this cohort.

Finally, the dynamic nature of EAT as a modifiable parameter must be emphasized. Previous studies have shown that EAT thickness decreases significantly with weight loss and improvement in insulin sensitivity. These results acquire particular relevance in the context of current pharmacologic approaches to obesity, including GLP-1 receptor agonists and dual incretin therapies, which achieve major reductions in visceral fat. Future longitudinal studies should assess whether decreased EAT, induced by these treatments, translates into measurable cardiovascular benefit and improved metabolic health.

This study has several limitations. First, the cross-sectional design precludes causal inferences regarding the relationship between epicardial adipose tissue thickness and metabolic or cardiovascular outcomes that should be validated by longitudinal studies to establish temporal associations and assess the predictive value of EAT for incident cardiometabolic and cardiovascular events. Second, the study population was restricted to patients with obesity from a single center in one geographic region, which may limit generalizability to other populations with different ethnic backgrounds, body composition distributions, or lower BMI ranges. Third, while dual-energy X-ray absorptiometry is an appropriate comparator for abdominal fat layers within the *Eco-Obesity* protocol, the absence of volumetric EAT assessment using computed tomography or magnetic resonance imaging to directly compare with sonographic EAT measurements represents a methodological limitation. Fourth, the subgroup undergoing comprehensive Eco-Obesity ultrasound evaluation was smaller (n=114) than the overall cohort, potentially reducing statistical power for certain subgroup and correlation analyses. Finally, muscle mass, muscle quality, and functional parameters related to sarcopenia and cardiac frailty (e.g. DEXA-derived lean mass, CT-based muscle indices, handgrip strength) were not systematically evaluated, precluding assessment of the emerging interplay between epicardial adipose tissue, skeletal muscle wasting, and adverse cardiovascular prognosis. Despite these limitations, the study presents important strengths, including the use of DEXA as a gold-standard comparator for body composition, the integration of multiple echocardiographic views of EAT with abdominal fat compartments, and the novel validation of ultrasound-based visceral fat quantification against established metabolic and anthropometric markers.

## 5 Conclusions

Given the high prevalence of pathological EAT thickness observed among individuals with obesity, routine ultrasonographic assessment of EAT should be integrated in a routinary ultrasound guided body composition analysis of ectopic fat depots (*Eco-Obesity* approach). EAT showed marked associations with metabolic parameters, particularly circulating triglycerides and glucose, supporting its relevance as a marker of metabolic dysfunction and insulin resistance. Hypertension and dyslipidemia were most prevalent among males demonstrating higher EAT values, reinforcing the intertwining cardiovascular and metabolic risk profiles. Additionally, the strong correlation between EAT and visceral adiposity measured by DEXA underscores the shared pathophysiological mechanisms underpinning ectopic fat deposition. However, not all visceral fat depots correlate with EAT. *Omental* and *peri-renal* fat showed nice correlations with EAT, whereas *pre-peritoneal* (included in DEXA visceral adiposity component) does not show any significant correlation.

Contemporary therapeutic strategies, including GLP-1 receptor agonists and dual analogs, have demonstrated efficacy in reducing visceral fat (*omental* and *peri-renal* fat in particular) (19, 20, 24), suggesting a potential for modifying epicardial fat as well. The integration of EAT assessment may therefore provide a novel means of monitoring therapeutic response and optimizing individualized management decisions. Future research should clarify whether increased epicardial fat mass could serve as a determining factor in selecting obesity pharmacotherapy and in predicting clinical outcomes, emphasizing the need for its evaluation within the broader context of obesity-related cardiovascular risk.

## Data Availability

The raw data supporting the conclusions of this article will be made available by the authors, without undue reservation.

## References

[1] Nadolsky K Garvey WT Agarwal M Bonnecaze A Burguera B Chaplin MD . American association of clinical endocrinology consensus statement: algorithm for the evaluation and treatment of adults with obesity/adiposity-based chronic disease - 2025 update. Endocr Pract. (2025) 31(11):1351–94. doi: 10.1016/j.eprac.2025.07.017, PMID: 40956256

[2] Gangitano E Barbaro G Susi M Rossetti R Spoltore ME Masi D . Growth hormone secretory capacity is associated with cardiac morphology and function in overweight and obese patients: A controlled, cross-sectional study. Cells. (2022) 11:2420. doi: 10.3390/cells11152420, PMID: 35954264 PMC9367721

[3] Rubino F Cummings DE Eckel RH Cohen RV Wilding JPH Brown WA . Definition and diagnostic criteria of clinical obesity. Lancet Diabetes Endocrinol. (2025) 13:221–62. doi: 10.1016/S2213-8587(24)00316-4, PMID: 39824205 PMC11870235

[4] Lee JH Kim JY Kim KM Lee JW Youn YJ Ahn MS . A prospective study of epicardial adipose tissue and incident metabolic syndrome: the ARIRANG study. J Korean Med Sci. (2013) 28:1762–7. doi: 10.3346/jkms.2013.28.12.1762, PMID: 24339706 PMC3857372

[5] Iacobellis G Ribaudo MC Assael F Vecci E Tiberti C Zappaterreno A . Echocardiographic epicardial adipose tissue is related to anthropometric and clinical parameters of metabolic syndrome: a new indicator of cardiovascular risk. J Clin Endocrinol Metab. (2003) 88:5163–8. doi: 10.1210/jc.2003-030698, PMID: 14602744

[6] Iacobellis G Corradi D Sharma AM . Epicardial adipose tissue: anatomic, biomolecular and clinical relationships with the heart. Nat Clin Pract Cardiovasc Med. (2005) 2:536–43. doi: 10.1038/ncpcardio0319, PMID: 16186852

[7] Sarin S Wenger C Marwaha A Qureshi A Go BDM Woomert CA . Clinical significance of epicardial fat measured using cardiac multislice computed tomography. Am J Cardiol. (2008) 102:767–71. doi: 10.1016/j.amjcard.2008.04.058, PMID: 18774004

[8] Chong B Jayabaskaran J Ruban J Goh R Chin YH Kong G . Epicardial adipose tissue assessed by computed tomography and echocardiography are associated with adverse cardiovascular outcomes: A systematic review and meta-analysis. Circ Cardiovasc Imaging. (2023) 16:e015159. doi: 10.1161/CIRCIMAGING.122.015159, PMID: 37192298

[9] Iacobellis G Willens HJ . Echocardiographic epicardial fat: a review of research and clinical applications. J Am Soc Echocardiogr. (2009) 22:1311–9; quiz 1417–8. doi: 10.1016/j.echo.2009.10.013, PMID: 19944955

[10] Iacobellis G Singh N Wharton S Sharma AM . Substantial changes in epicardial fat thickness after weight loss in severely obese subjects. Obes (Silver Spring). (2008) 16:1693–7. doi: 10.1038/oby.2008.251, PMID: 18451775

[11] Cuatrecasas G de Cabo F Coves MJ Patrascioiu I Aguilar G March S . Ultrasound measures of abdominal fat layers correlate with metabolic syndrome features in patients with obesity. Obes Sci Pract. (2020) 6:660–7. doi: 10.1002/osp4.453, PMID: 33354344 PMC7746969

[12] Iacobellis G . Epicardial fat links obesity to cardiovascular diseases. Prog Cardiovasc Diseases. (2023) 78:27–33. doi: 10.1016/j.pcad.2023.04.006, PMID: 37105279

[13] Wang G Luo Y Yang T Huang J Li J Liu Y . Association of waist-to-height ratio with all-cause and obesity-related mortality in adults: a prospective cohort study. Front Nutr. (2025) 12:1614347. doi: 10.3389/fnut.2025.1614347, PMID: 40860487 PMC12375492

[14] American Diabetes Association . 2. Classification and diagnosis of diabetes: standards of medical care in diabetes-2018. Diabetes Care. (2018) 41:S13–27. doi: 10.2337/dc18-S002, PMID: 29222373

[15] American Diabetes Association . 9. Cardiovascular disease and risk management: standards of medical care in diabetes-2018. Diabetes Care. (2018) 41:S86–104., PMID: 29222380 10.2337/dc18-S009

[16] Sacks HS Fain JN . Human epicardial adipose tissue: a review. Am Heart J. (2007) 153:907–17. doi: 10.1016/j.ahj.2007.03.019, PMID: 17540190

[17] Iacobellis G Willens HJ Barbaro G Sharma AM . Threshold values of high-risk echocardiographic epicardial fat thickness. Obes (Silver Spring). (2008) 16:887–92. doi: 10.1038/oby.2008.6, PMID: 18379565

[18] Asano T Kubota N Koizumi N Itani K Mitake T Yuhashi K . Novel and simple ultrasonographic methods for estimating the abdominal visceral fat area. Int J Endocrinol. (2017) 2017:8796069. doi: 10.1155/2017/8796069, PMID: 29093737 PMC5585558

[19] Cuatrecasas G Calbo M Rossell O Dachs L Aguilar-Soler G Coves MJ . Effect of liraglutide in different abdominal fat layers measured by ultrasound: the importance of perirenal fat reduction. Obes Facts. (2024) 17:347–54. doi: 10.1159/000538996, PMID: 38643760 PMC11299965

[20] Cuatrecasas G De Cabo F Coves MJ Patrascioiu I Aguilar G Cuatrecasas G . Dapagliflozin added to metformin reduces perirenal fat layer in type 2 diabetic patients with obesity. Sci Rep. (2024) 14:10832. doi: 10.1038/s41598-024-61590-6, PMID: 38734755 PMC11088615

[21] Komici K Bencivenga L Arganese C Rengo G Guerra G . The relationship between muscle strength and epicardial fat in healthy adults. Exp Gerontol. (2024) 192:112447. doi: 10.1016/j.exger.2024.112447, PMID: 38692441

[22] Janovska P Melenovsky V Svobodova M Havlenova T Kratochvilova H Haluzik M . Dysregulation of epicardial adipose tissue in cachexia due to heart failure: the role of natriuretic peptides and cardiolipin. J Cachexia Sarcopenia Muscle. (2020) 11:1614–27. doi: 10.1002/jcsm.12631, PMID: 33084249 PMC7749591

[23] Walpot J Van Herck P Van de Heyning CM Bosmans J Massalha S Inácio JR . Computed tomography measured epicardial adipose tissue and psoas muscle attenuation: new biomarkers to predict major adverse cardiac events and mortality in patients with heart disease and critically ill patients. Part II: Psoas Muscle area density Anaesthesiol Intensive Ther. (2023) 55:243–61. doi: 10.5114/ait.2023.130922, PMID: 38084569 PMC10691466

[24] Myasoedova VA Parisi V Moschetta D Valerio V Conte M Massaiu I . Efficacy of cardiometabolic drugs in reduction of epicardial adipose tissue: a systematic review and meta-analysis. Cardiovasc Diabetol. (2023) 22:23. doi: 10.1186/s12933-023-01738-2, PMID: 36721184 PMC9890718

